# Evolutionary dynamics of ten novel *Gamma-PVs*: insights from phylogenetic incongruence, recombination and phylodynamic analyses

**DOI:** 10.1186/s12864-019-5735-9

**Published:** 2019-05-14

**Authors:** Alltalents T. Murahwa, Fredrick Nindo, Harris Onywera, Tracy L. Meiring, Darren P. Martin, Anna-Lise Williamson

**Affiliations:** 1 Division of Medical Virology, Department of Pathology, Faculty of Health Sciences, University of Cape Town, Cape Town, 7925 South Africa; 2 Institute of Infectious Disease and Molecular Medicine, University of Cape Town, Cape Town, South Africa; 3 Division of Computational Biology, Department of Integrative Biomedical Sciences, Faculty of Health Sciences, University of Cape Town, Cape Town, 7925 South Africa; 4 SAMRC Gynaecological Cancer Research Centre, Faculty of Health Sciences, University of Cape Town, Cape Town, South Africa

**Keywords:** Human papillomavirus, Gamma-PVs, Most recent common ancestor, Phylogenetic incongruence, Recombination, Molecular divergence

## Abstract

**Background:**

Human papillomaviruses (HPVs) are genetically diverse, belonging to five distinct genera: Alpha, Beta, Gamma, Mu and Nu. All papillomaviruses have double stranded DNA genomes that are thought to evolve slowly because they co-opt high-fidelity host cellular DNA polymerases for their replication. Despite extensive efforts to catalogue all the HPV species that infect humans, it is likely that many still remain undiscovered. Here we use the sequences of ten novel *Gammapapillomaviruses* (*Gamma-PVs*) characterized in previous studies and related HPVs to analyse the evolutionary dynamics of these viruses at the whole genome and individual gene scales.

**Results:**

We found statistically significant incongruences between the phylogenetic trees of different genes which imply gene-to-gene variation in the evolutionary processes underlying the diversification of Gamma-PVs. We were, however, only able to detect convincing evidence of a single recombination event which, on its own, cannot explain the observed incongruences between gene phylogenies. The divergence times of the last common ancestor (LCA) of the Alpha, Beta, Mu, Nu and Gamma genera was predicted to have existed between 49.7–58.5 million years ago, before splitting into the five main lineages. The LCA of the Gamma*-PVs* at this time was predicted to have existed between 45.3 and 67.5 million years ago: approximately at the time when the simian and tarsier lineages of the primates diverged.

**Conclusion:**

Consequently, we report here phylogenetic tree incongruence without strong evidence of recombination.

## Background

Human papillomavirus (HPV) is a member of the *Papillomaviridae* family [1, 2] which was once part of the larger family of *Papovaviridae* which was split into *Polyomaviridae* and *Papillomaviridae* by the International Committee on Taxonomy of Viruses (ICTV) [3]. There are currently 29 genera in the *Papillomaviridae* family named according to the Greek alphabet from alpha to omega and following exhaustion of the alphabet the term dyo-(Greek for second time) coined to accommodate the extra genera e.g. dyo-*deltapapillomaviruses* [1]. HPVs are distributed over 5 genera (Alpha, Beta, Gamma, Mu and Nu). The other papillomavirus (PV) genera are from other mammals (20), birds (3) and reptiles (1) [1]. Below the genus level are species and below the species level are types [2]. The ICTV is responsible for nomenclature of viruses down to species level, and below species level, the International HPV Reference Centre in Stockholm Sweden assigns unique HPV type numbers after the complete genome has been sequenced, cloned and confirmed by the Centre [4]. The recognition of a novel HPV type by the International HPV Reference Centre and the scientific community is based on availability of the full cloned genome, with the L1 gene sequence greater than 10% different or < 90% similar from any previously described type [1].

HPVs are thought to evolve slowly because they replicate by co-opting high fidelity host cellular DNA polymerases that have an error rate of about 4.3 × 10^− 5^ substitutions per nucleotide site per year [5]. It is therefore generally assumed that HPVs have co-evolved with their hosts [6–8]. However, several host factors may affect HPV evolutionary rates over time; for example, the cellular protein APOBEC3 cytidine deaminase may result in nucleotide compositional biases that contribute to cytosine containing codons accruing more frequently that thiamine containing codons. Selection pressures due to cellular or humoral immune responses may also differ between genes and result in these genes displaying different substitution rates. Further, the cellular polymerases of different host species may differ in their degree of fidelity such that virus lineages infecting different hosts might display different substitution rates [9].

It has been estimated that PVs appeared about 250–150 million years ago [10] in the Mesozoic age (age of reptiles/dinosaurs) [6]. Whereas the most recent common ancestor (MRCA) of the Alpha, Beta, Gamma, Mu and Nu HPVs has been inferred to have existed between 30 and 50 MYA [11], the MRCA of the present day *Gamma-PVs* has been inferred to have existed between 15 and 30 MYA [11]. The evolutionary rate of PVs has been estimated from feline-PVs to be about 1.95 × 10^− 8^ nucleotide substitutions per site per year [12].

The discovery of numerous new PVs using deep sequencing methods has begun to shed further light on the evolutionary history of this virus family. However, unravelling the evolutionary history of these viruses is potentially complicated by both inter-gene phylogenetic incongruence and recombination [13]. It has been observed that the nucleotide and encoded amino acid sequences of the E and L genes have evolved slightly differently in terms of evolutionary rate and selection pressures [14, 15]: a factor that could contribute to phylogenetic incongruence between the E and L gene trees. As a consequence of this, no single gene tree will accurately and adequately represent the evolutionary history of complete papillomavirus genomes [13]. Recombination events between different PVs may provide an additional explanation for gene-to-gene phylogenetic incongruence.

The biological plausibility of PV recombination is occasioned by the genetic diversity of PVs and the high frequencies of observed HPV co-infections [16]. However, the study of PV recombination has been hampered by technical difficulties associated with the accurate alignment of highly diverse PV gene sequences [17]. One of the most commonly used approaches to recombination detection is the use of the various recombination analysis tools implemented within the RDP4 software package [18]. During recombination detection, RDP4 rigorously tests the quality of sequence alignments to guard against the detection of false-positive recombination signals that arise due to sequence misalignment [19].

We report here on the use of sequences of ten novel *Gamma-PVs* characterized in a previous studies [20, 21], and related HPVs to analyse the evolutionary dynamic of these viruses at the whole genome and individual gene levels. Specifically, we use phylogenetic tree incongruence tests to identify incongruence and direct recombination tests to determine whether observed phylogenetic incongruences might be linked to recombination. We further use the novel sequences to estimate the likely times of the MRCA of the *Gamma-PVs*.

## Methods

### Source of sequence data

Sequences were downloaded from the Papillomavirus episteme (PAVE) database (https://pave.niaid.nih.gov accessed on 27/01/2018). The sequences for HPV211-HPV216 and HPV219-HPV222 were obtained from our group [20, 21] . The novel HPV DNA sequences were deposited in Genbank under the following accession numbers: HPV211 MF509816, HPV212 MF509817, HPV213 MF509818, HPV214 MF509819, HPV215 MF509820, and HPV216 MF509821. The GenBank accession numbers for HPV219, HPV220, HPV221, HPV222 genomes are MH172376, MH172377, MH172378, MH172379, respectively. PVs are classified by the International Committee on Taxonomy of Viruses (ICTV) into distinct species, but the nomenclature of types can be done by specific working groups. Some of the viruses used in this study are pending classification by the ICTV, but were provisionally grouped into specific genera and types by the HPV reference centre in Sweden (http://www.nordicehealth.se/hpvcenter/reference_clones/).

### Phylogenetic incongruence testing

#### The Shimodaira-Hasegawa test [22] using W-IQ-TREE [23]

We used clustal alignments [24–26] of E1, E2, E4, E7, L1 and L2 HPV genes from 80 Gamma-HPVs (including the ten novel types) to compute the log-likelihoods of phylogenetic trees in W-IQ-TREE, which is a fast online phylogenetic tool for maximum likelihood analysis (http://iqtree.cibiv.univie.ac.at) [23]. The tool test tree topology estimates model parameters such as substitution rates and optimizes tree branch lengths to lessen computational usage. We used default settings of the W-IQ-Tree, including best fit model [27] and ultra-fast bootstrap analysis (1000 alignments) [28] to run tree topology analysis including the Kishino-Hasegawa (KH) test [29], Shimodaira-Hasegawa (SH) test [22] and approximately unbiased (AU) test [30] to test if there is a difference in evolutionary patterns amongst the different HPV genes.

### Recombination analysis

Eighty complete Gamma-PV genomes that are representative of all currently known *Gamma-PVs* were obtained from the PAVE database, including the ten novel HPV types recently discovered and genomically characterised by our group [20, 21] . All genomes were linearized at the first nucleotide position of their E6 genes except for Gamma species 6 viruses that lack E6 and for which the start was shifted to the first nucleotide of their E7 genes. We then constructed an alignment containing the 80 *Gamma-PVs* using MUSCLE*.* This alignment was analysed using RDP v4.95 [18] (with default settings) which implements analysis of recombination using several methods: RDP [18], BOOTSCAN [31], CHIMAERA [32], GENECONV [33], MAXIMUM X^2^ [34] and SISCAN [35].

### Construction of a time-scaled HPV phylogeny

We sought to infer the probable divergence times of our newly characterised HPV types from currently known HPVs. The complete L1 nucleotide sequences from 214 PV sequences were selected for analysis (Table 1). Two avian PVs: FcPV (*Fringilla coelebs*, the common chaffinch), PePV (*Psittacus erithacus*, the grey parrot); one turtle PV: CcPV1 (*Caretta caretta*, the loggerhead turtle) and one bovine PV (BPV1) were also included in the analysis as outgroups. We performed a Bayesian evolutionary molecular clock analysis using BEAST v1.8.4 [36] with a GTR + I + G nucleotide substitution model and an uncorrelated lognormal relaxed clock model. A fixed mean substitution rate for HPVs was applied based on estimated evolutionary rates inferred from prior studies that investigated the divergence times of feline PVs (1.95 × 10^− 8^ nucleotide substitutions per site per year) [12]. The Markov Chain Monte Carlo (MCMC) analysis was run for 100,000 million generations with sampling every 10,000 generations. The final MCMC sampling chains were visually assessed for convergence and good mixing using Tracer v1.7.1 [37]. A Maximum Clade Credibility (MCC) tree was generated after discarding the first 1000 trees that were obtained prior to the burn-in period of the chains.

**Table 1:** Summary of Analysis Performed and sequence dataset used

Type of analysis	Sequence data set used
Shimodaira-Hasegawa test	80 whole genomes of currently known Gamma-HPVs
Recombination analysis	80 whole genomes of currently known Gamma-HPVs
Construction of a time-scaled HPV phylogeny	214 L1 nucleotide sequences of mostly HPV sequences and 2 avian PVs, one turtle PV and one bovine PV

## Results

### Phylogenetic incongruence among novel *Gamma-PVs* gene trees

To determine if the phylogenetic trees for different Gamma-PV genes were congruent, we used a more conclusive test, the SH test [22]. The null hypothesis of the SH test states that the difference between trees (branch length, topology or likelihoods) is zero. The observed differences (deltaL values) are significantly greater than zero and the null hypothesis was rejected, thus declaring that the trees are significantly different i.e. incongruent (*p* < 0.05). Table 2 shows the results of the SH test using W-IQ-Tree, indicating that there is substantial phylogenetic incongruence between the late and early genes of Gamma-PVs as shown by the *p*-values (p-SH).

**Table 2:** Shimodaira-Hasegawa test for incongruence

E1 as reference tree								
Tree	deltaL	bp-RELL	p-KH	p-SH	p-WKH	p-WSH	c-ELW	p-AU
E1	0	1	1	1	1	1	1	0.999
E2	182.16	0	0	0.008	0	0	7.3e-33	0.000747
E4	639.14	0	0	0	0	0	3.8e-203	0.000845
E7	1048.70	0	0	0	0	0	0	1.8e-58
L1	381.21	0	0	0	0	0	3.8e-104	4.2e-05
L2	219.47	0	0	0.002	0	0	1.2e-40	2.4e-09
E2 as reference tree								
Tree	deltaL	bp-RELL	p-KH	p-SH	p-WKH	p-WSH	c-ELW	p-AU
E1	137.43	0	0	0.023	0	0	1.3e-27	8.3e-05
E2	0	1	1	1	1	1	1	1
E4	288.35	0	0	0	0	0	1.3e-75	2.8e-07
E7	904.92	0	0	0	0	0	3.4e-299	6.7e-97
L1	273.21	0	0	0	0	0	5.9e-70	5.7e-44
L2	224.96	0	0	0	0	0	1.8e-60	5.1e-07
E4 as reference tree								
Tree	deltaL	bp-RELL	p-KH	p-SH	p-WKH	p-WSH	c-ELW	p-AU
E1	79.74	0	0	0.012	0	0.004	0.000433	0.0217
E2	64.99	0.02	0.003	0.02	0.003	0.016	0.00211	0.104
E4	0	0.998	0.997	1	0.997	1	0.997	0.992
E7	311.86	0	0	0	0	0	8.7e-85	5.8e-114
L1	130.15	0	0	0	0	0	1.2e-23	6.4e-35
L2	84.83	0	0	0.004	0	0	1.1e-10	0.00483
E7 as reference tree								
Tree	deltaL	bp-RELL	p-KH	p-SH	p-WKH	p-WSH	c-ELW	p-AU
E1	59,374	0.004	0.016	0.017	0.016	0.064	0.00429	0.0117
E2	71.02	0.001	0.007	0.007	0.007	0.031	0.000919	0.00182
E4	138.43	0	0	0	0	0	1.78e-20	0.014
E7	0	0.946	0.952	1	0.952	0.991	0.944	0.945
L1	39.42	0.0041	0.048	0.119	0.048	0.189	0.043	0.118
L2	59.86	0.008	0.022	0.025	0.022	0.063	0.00759	0.0321
L1 as reference tree								
Tree	deltaL	bp-RELL	p-KH	p-SH	p-WKH	p-WSH	c-ELW	p-AU
E1	188.69	0	0	0	0	0	1.3e-25	7.6e-05
E2	218.51	0	0	0	0	0	4.1e-40	6.6e-48
E4	473.21	0	0	0	0	0	1.6e-133	0.000267
E7	899.66	0	0	0	0	0	3.5e-301	5.98e-65
L1	0	1	1	1	1	1	1	1
L2	149.83	0	0	0.005	0	0	9.9e-301	1.23e-10
L2 as reference tree								
Tree	deltaL	bp-RELL	p-KH	p-SH	p-WKH	p-WSH	c-ELW	p-AU
E1	226.34	0	0	0	0	0	2.4e-51	1.3e-52
E2	220.29	0	0	0	0	0	1.8e-50	8.4e-12
E4	478.78	0	0	0	0	0	1.5e-149	4.2e-06
E7	1087.1	0	0	0	0	0	0	0.00124
L1	229.72	0	0	0	0	0	1.3e-42	2.01e-13
L2	0	1	1	1	1	1	1	1

deltaL: logL difference from the maximal logl in the set

bp-RELL: bootstrap proportion using RELL method [38]

p-KH: *p*-value of one sided [29]

p-SH: *p*-value of Shimodaira-Hasegawa test [22]

p-WKH: p-value of weighted KH test

p-WSH: *p*-value of weighted SH test

c-ELW: Expected Likelihood Weight [39]

p-AU: p-value of approximately unbiased (AU) test [30]

### Recombination analysis

The *Gamma-PV* whole genome alignments contained a total of only three plausible recombination events, namely: events 1, 2 and 3. These events were all detectable by two or more different recombination detection methods with a *p*-value cut-off < 0.05. However, event 1 and event 2 had no phylogenetic support and were therefore disregarded. Event 3 suggests that HPV4 and its near relatives all share evidence of the same ancestral recombination event [21] Table 3.

**Table 3:** Recombination event 3 and other potential recombination signals in whole genome *Gamma-PVs*

Estimated Break point positions				Recombinant (Gamma-species)	Parent sequence		Evidence (method with *P* value < 0.05)	Intra-species or inter species recombination
In Alignment		In Genbank sequence			Minor (Gamma-species)	Major (Gamma-species)		
4446	8018	3631	6426	^HPV4 (γ-1)	HPV130 (γ-10)	Unknown (HPV162)(γ-19)	M,C,S, 3S	Inter-species
				HPV163[T]				
				HPV173[T]				
				HPV205[T]				
				HPV158[T]				
				HPV95[T]				
				HPV65[T]				

N.B Only event 3 is shown in Table 2, all the potential recombinant *Gamma-PVs* for this event are shown in the recombinant column and the proposed major and minor parents

^ = The recombinant sequence may have been misidentified (one of the identified parents might be the recombinant

Minor Parent = Parent contributing the smaller fraction of sequence

Major Parent = Parent contributing the larger fraction of sequence

Unknown = only one parent and a recombinant need be in the alignment for a recombination event to be detectable the sequence listed as unknown was used to infer the existence of a missing parental sequence

[T] Sequences with trace evidence of the same recombinant event

### The time-scale of *Gamma-PVs* evolution

A fixed mean substitution rate for HPVs was applied based on estimated evolutionary rates inferred from of feline PVs (1.95 × 10^− 8^ nucleotide substitutions per site per year) [19]. The divergence times of the MRCA of HPV was predicted to have occurred 53.9 MYA (95% HPD 49.7–58.5), before splitting into the five main potential ancestors (Alpha, Beta, Mu, Nu and Gamma genera). The MRCA of the present-day *Gamma-PVs* was predicted to have occurred approximately 49.8 MYA (95% HPD 45.3–67.5). The novel HPV 212 was predicted to have diverged from its closest relative, HPV144, about 7.6 MYA (95% HPD 5.2–10.4), divergence times from the MRCA of the remaining nine novel *Gamma-PVs* are shown in Table 4. The predictions lie between 7.6 to 19.9 MYA.

**Table 4:** Mean divergence time of 10 novel HPVs from other gamma species or closest relative

HPV types	Gamma-Species	Posterior	Mean Divergence Time (95% Highest Posterior Density) from MRCA
HPV211	8	1	19.9 (16.8–23.1) from all other Gamma-8 species HPV types
HPV212	17	1	7.6 (5.2–10.4) from its closest relative HPV144
HPV213	13	1	5.4 (4.0–7.1) from its closest relative HPV219
HPV214	6	1	17 (12.8–20.7) from closest relatives HPV108 and HPV103
HPV215	9	1	11.3 (9.2–13.7) from closest relative HPV216 and HPV129
HPV216	9	1	8.3 (6.2–10.6) from closest relative HPV129
HPV219	13	1	5.4 (4.0–7.1) from its closest relative HPV219
HPV220	17	1	19.9 (15.1–23.0) from all other Gamma-17 species HPV types
HPV221	10	1	8.3 (5.8–11.5) from closest relative HPV142
HPV222	19	1	19.2 (15.7–23.0) from all other Gamma-19 species HPV types

It has been reported that between 10 and 20 MYA, several hominoid precursors lived in Africa, Europe and Asia [40] and the timing and spatiotemporal patterning of the disappearance of Neanderthal human precursors has only been radiocarbon dated to a limit of 50,000 years ago [41]. Thus, our MRCA prediction suggests that all the current HPV species diverged from what are currently their nearest relatives before the origin of humans.

**Fig. 1:**
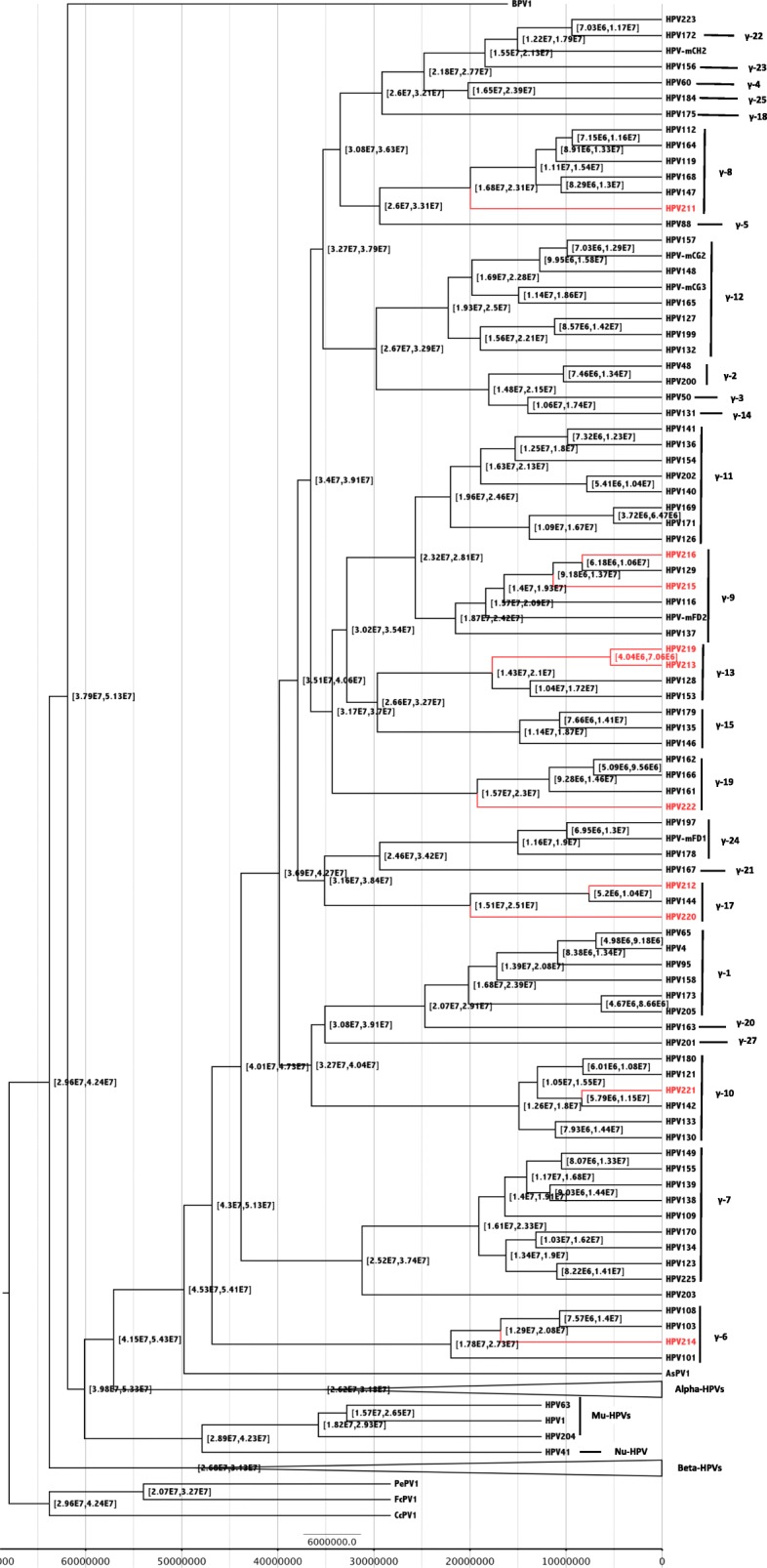
Molecular divergence times of *PVs.* A fixed mean substitution rate for HPVs was applied based on estimated evolutionary rates inferred from a study that investigated the times to the MRCA based of feline papillomaviruses (1.95 × 10^− 8^ nucleotide substitutions per site per year) [12]. Classification was based on [1, 2]. Posterior support values and exact divergence estimates in million years (with 95% highest posterior density) for the nodes corresponding to the 10 HPV types are presented in Table 2, the novel types are indicated in red

The MRCA of the Gamma-6 species was predicted to have existed 22 MYA (95% HPD 17.7–27.3) earlier than that of most other *Gamma-PVs* species*.* The MRCA of all other Gamma-species viruses and the Gamma-6 viruses was predicted to have occurred 46.8 MYA (95% HPD 42.9–51.3). The rest of the node divergence times are shown in Fig. 1 with the 95% highest posterior densities.

## Discussion

### Phylogenetic incongruence and recombination

An analysis of phylogenetic trees generated from different genes of the ten novel South African *Gamma-PVs* and their closest known relatives indicated differences, ranging from slight to major, between the branch lengths and branching orders of phylogenetic trees constructed from different genes. Differences between internal branches of phylogenetic trees constructed using different parts of the same HPV genomes have been described extensively elsewhere [6, 42, 43]. These differences could imply the occurrence of recombination, as has been previously reported [44]. Different *PV* genes have been shown to have different evolutionary rates, with an overall rate of 1.95 × 10^− 8^ and a range of 1.76 × 10^− 8^ to 2.69 × 10^− 8^ substitutions/site/year [13]. The PV genome has a modular nature, which is evident today in the different evolutionary rates of the different genes, the new genes E5, E6 and E7 encoding oncoproteins diverge faster than the old four genes E1, E2, L2 and L1 [15]. The four old proteins are by themselves able to fulfil the basic tasks of replication, regulation, stabilisation and viral DNA packaging leading to vegetative release of viral progeny from host cells [45]. The acquisition of the new oncogenes has introduced two PV phylogenies, high risk Alpha-PVs cluster together according to the phylogeny of these oncogenes (E5, E6, E7) but they do not cluster together according to the phylogeny of the capsid proteins (L1 and L2) [42]. Additionally, the number of mutations are higher in the new oncogenes than in most of the *PV* genes [42]. It is thus proposed that the history of PVs took place in different stages, the first stage represents the initial appearance of a prototype-PV with the cardinal genes (E1, E2, L2 and L1) found in all *PVs*. Then second stage involved further divergent evolutionary processes that led to the acquisition of the E5, E6 and E7 oncoproteins, and these newly acquired proteins evolved about two times faster than the core region of the genome [15]. It has been observed that the genes and encoded amino acid sequences of viral early and late proteins have evolved differently in terms of evolutionary rate and selection pressure [14, 15], and hence the incongruence between early and late trees. *PV* recombination events may provide a clue to the phylogenetic incongruence, but recombination is not the only explanation for this incongruence.

We did not, however find strong evidence of recombination among *Gamma-HPV* types. From the 80 full *Gamma-PVs* (70 known HPV types and the ten novel types) included in the analysis only one strongly supported recombination event was detected. Recombination in *Gamma-PVs* has been reported elsewhere [46]. In that study, seven potential recombination events were reported using separate analyses of individual genes (rather than analysing full genomes). We detected few *Gamma-PVs* recombination events. Varsani et al. (2006) [19] reported 529 potential recombination events, yet only ten were true events. The phenomenon is not exclusive to *Gamma-PVs*, in *Alphapapillomaviruses* recombination events have been well described [16, 47].

The difficulty of detecting recombination events in *PVs* relates to the technical difficulty of aligning highly divergent DNA sequences [6, 16, 19]. Another factor is that most recombination detection methods are only designed to detect recombination events when one or both of the parental sequences have close relatives represented in the dataset being analysed [13]. Therefore, as more PVs are discovered it becomes more likely that sequences closely related to the parents of recombinants will be included in recombination analyses. We also only included *Gamma-PVs*, in our recombination analysis dataset and our analysis was therefore only powered to detect for evidence of intra-genus recombination. However, this is probably not a major issue since the only convincing evidence of recombination in *PVs* that has so far been published has been between *PVs* in the same genus [47]. We have previously reported that the ten novel *Gamma-PVs* are under no positive selection pressure but rather purifying selection (dn/ds < 1) ([21].

It has been shown elsewhere that different *PV* genes are under different selection pressures [48] and also that different genes have different evolutionary rates ranging between 2 × 10^− 8^ and 5 × 10^− 9^ substitutions per site per year [12, 44]. Thus, no single gene tree will accurately represent the evolutionary history of entire *PV* genomes [13].

Consequently, we report here phylogenetic tree incongruence with no evidence of recombination. It has been proposed that in such scenarios there is convergent as opposed to divergent evolution [49]. Convergent evolution can be defined as the independent evolution of similar features or characteristics in species of different lineages.

### Temporal evolution within *Gamma-PVs*

We show here that the MRCA of HPV was predicted to have occurred about 50–60 MYA. This is comparable to work done by Chen et al. (2007) [11], and that the MRCA of the *Gamma-PVs* existed about 45–67 (49.8) MYA. Further, we were able to show that within the *Gamma-PV* genus, the Gamma-6 species split from the rest of all the other Gamma species about 43–51 (46.8) MYA, while Van Doorslaer and Mcbride (2016) [50] showed that Gamma-6 species last shared a common ancestor with other *Gamma-PVs* around 60 MYA. Further, we showed that the MRCA of the Gamma-6 species occurred about 22 MYA, which concurs with Van Doorslaer and Mcbride (2016) [50] who reported that the Gamma-6 species MRCA existed 23.4 MYA. Therefore, we hypothesise that the Gamma-6 species lost the E6 gene between 20 and 60 MYA. This suggests that E6 was lost before the evolution of hominoid primates between 10 and 20 MYA [40]. This implies two things: 1) that viruses lacking E6 may infect old world and new world monkeys, suggesting that it could be productive to hunt for these viruses in primates, and 2) that the E6-minus viruses co-evolved with their hosts over a long enough period of time for us to have been able to isolate them from current humans (assuming papillomaviruses species specificity). Chen et al. (2007) [11] had previously predicted the loss of the E6 gene to have occurred about 15–30 MYA, the estimate was based only on the L1 open reading frame (ORF) of nine divergent papillomaviruses. Here, we have estimated this date using 214 papillomavirus L1 nucleotide sequences from all the genera containing HPV sequences.

PVs lacking E6 have also been described in parrots (PePV1), donkeys (EaPV1) and bovines [51]. Presently, 7 known human PVs of the Gamma-6 species lack the E6. The size of E6 (mean 253.5 nt) in the genomes of PVs infecting birds and turtles is about half the size that it is in mammal-infecting PV genomes (mean 438 nt) [51]. The larger size of the mammalian PV E6 accommodates a second E6 zinc finger binding motif domain [52]: a domain that was possibly the result of duplication of an ancestral E6 motif [53]. E6 mediated p53 degradation has been described as one of the hallmarks of HPV mediated carcinogenesis, and the presence of this double motif may explain the increased likelihood of HPVs in causing cancer compared to PVs infecting birds and turtles.

*Gamma-PVs* lack an E5 gene, which is located between the early genes and the late genes and is thought to have evolved originally from a non-coding region that was integrated between the early and the late genes of an ancestral sequence belonging to the *Alphapapillomaviruses* lineage [51]. Willem et al. also suggest that integration of E5 in this region promoted an adaptive radiation which yielded E6 and E7 proteins capable of degrading tumour suppressor proteins and facilitating carcinogenesis [51]. This is supported by the fact that 1) E6 and E7 proteins in Alpha PVs (together with E5) have greater oncogenic potential as classified by IARC as compared to the E6 and E7 of Gamma-PVs (that lack E5) and 2) E5 has an evolutionary rate that is approximately twice that of the remainder of the PV genome [54]. The integration of the E5 ORF was predicted to have occurred in an ancestral virus that existed 30–60 MYA (in the Cenozoic era) which eventually gave rise to the Alpha. Mu and Nu lineages; each of which has different cell tropisms and clinical manifestations [6]. Willem et al. [51] inferred the appearance of the E5 oncogene occurred 53–58 MYA: well within the range of that predicted by Bravo et al. [6].

We also reported the acquisition of E10 in HPV214 [21], which we hypothesised coincided with E6 loss as previously reported [55]. We speculate that if the loss of E6 occurred 20–60 MYA then E10 was acquired a few million years later or that the loss and gain might have occurred concurrently as a modification of E6 to E10. This is supported by the fact that the E10 ORF overlaps with the more conserved portion of the E6 scar [50].

We report divergence times from 7.6 to 20 MYA with most lying well within the origin times of many other known PVs. However, HPV211 of the Gamma-8 species branches earlier from the other five HPV types in this species, i.e., the MRCA of HPV211 and the other 5 Gamma-8 species types is predicted to have occurred 20 MYA. This implies that HPV211 is closer to the ancestral or parental sequence of Gamma-8 species compared to HPV112, HPV119, HPV168, HPV147, and HPV164, and hence, it is more likely to be major/minor parent than it can be a recombinant. The MRCA of HPV222 and the other 3 members of the Gamma-19 species is 19.2 MYA. HPV222 branches from HPV161, HPV162 and HPV166 earlier than the others, also making it closer to the ancestral or parental sequence of the Gamma-19 species.

## Conclusion

In this work, we report the evolutionary characterisation of *Gamma-PVs* including that of the novel HPV types. To get a deeper insight into the evolutionary processes that may influence the diversification of *Gamma-PVs*, we explored phylogenetic incongruences among different genes of the novel types, attempted to discover potential recombination events between all known *Gamma-PVs*, and also estimated the time scale for *Gamma-PVs* evolution. Consequently, we report here phylogenetic tree incongruence without strong evidence of recombination.

## Abbreviations

BPV1: *Bovine PV*
CcPV1: *Caretta caretta PV*
FcPV: *Fringilla coelebs PV*
*Gamma-PVs*: *Gammapapillomaviruses*
HPV: Human papillomavirus
ICTV: International Committee on Taxonomy of Viruses
KH: Kishino-Hasegawa
LCA: Last common ancestor
MCC: Maximum Clade Credibility
MCMC: Markov Chain Monte Carlo
MRCA: Most recent common ancestor
MYA: Million years ago
PePV: *Psittacus erithacus PV*
PV: Papillomavirus
SH: Shimodaira-Hasegawa

## References

[1] Bernard HU (2010). Classification of papillomaviruses (PVs) based on 189 PV types and proposal of taxonomic amendments. Virology.

[2] de Villiers EM (2004). Classification of papillomaviruses. Virology.

[3] vanRegenmortel, ICTV. ictv report, 2002.

[4] Mühr LSA, Eklund C, Dillner J (2018). Towards quality and order in human papillomavirus research. Virology.

[5] Korona DA, Lecompte KG, Pursell ZF (2011). The high fidelity and unique error signature of human DNA polymerase epsilon. Nucleic Acids Res.

[6] Bravo IG, Felez-Sanchez M (2015). Papillomaviruses: viral evolution, cancer and evolutionary medicine. Evol Med Public Health.

[7] Chen Z (2009). Evolutionary dynamics of variant genomes of human papillomavirus types 18, 45, and 97. J Virol.

[8] Dube Mandishora RS (2018). Intra-host sequence variability in human papillomavirus. Papillomavirus Res.

[9] de Oliveira CM (2015). High-level of viral genomic diversity in cervical cancers: a Brazilian study on human papillomavirus type 16. Infect Genet Evol.

[10] Smelov V (2018). Beta and gamma human papillomaviruses in anal and genital sites among men: prevalence and determinants. Sci Rep.

[11] Chen Z (2007). Human papillomavirus (HPV) types 101 and 103 isolated from cervicovaginal cells lack an E6 open reading frame (ORF) and are related to gamma-papillomaviruses. Virol J.

[12] Rector A (2007). Ancient papillomavirus-host co-speciation in Felidae. Genome Biol.

[13] Van Doorslaer K (2013). Evolution of the papillomaviridae. Virology.

[14] Harari A, Chen Z, Burk RD (2014). Human papillomavirus genomics: past, present and future. Curr Probl Dermatol.

[15] García-Vallvé S, Alonso Á, Bravo IG (2005). Papillomaviruses: different genes have different histories. Trends Microbiol.

[16] Angulo M, Carvajal-Rodriguez A (2007). Evidence of recombination within human alpha-papillomavirus. Virol J.

[17] Posada D, Crandall KA (2001). Evaluation of methods for detecting recombination from DNA sequences: computer simulations. Proc Natl Acad Sci.

[18] Martin D, Rybicki E (2000). RDP: detection of recombination amongst aligned sequences. Bioinformatics.

[19] Varsani A (2006). Evidence of ancient papillomavirus recombination. J Gen Virol.

[20] Murahwa AT, et al. Complete genome sequences of four novel human Gammapapillomavirus types, HPV-219, HPV-220, HPV-221, and HPV-222, isolated from penile skin swabs from south African men. Genome Announc. 2018;6(25). 10.1128/genomeA.00584-18 PMC6013601 29930074

[21] Murahwa, A.T., et al., Discovery, characterisation and genomic variation of six novel gammapapillomavirus types from penile swabs in South Africa*.* Papillomavirus Research, 2019. epub ahead of print(10.1016/j.pvr.2019.02.005). 10.1016/j.pvr.2019.02.005 PMC6416656 30844514

[22] Shimodaira H, Hasegawa M (1999). Multiple comparisons of log-likelihoods with applications to phylogenetic inference. Mol Biol Evol.

[23] Trifinopoulos J (2016). W-IQ-TREE: a fast online phylogenetic tool for maximum likelihood analysis. Nucleic Acids Res.

[24] Sievers F (2011). Fast, scalable generation of high-quality protein multiple sequence alignments using Clustal omega. Mol Syst Biol.

[25] Li W (2015). The EMBL-EBI bioinformatics web and programmatic tools framework. Nucleic Acids Res.

[26] McWilliam, H., et al., Analysis Tool Web Services from the EMBL-EBI Nucleic Acids Res, 2013. 41(Web Server issue): p. W597–600. 10.1093/nar/gkt376 PMC3692137 23671338

[27] Kalyaanamoorthy S (2017). ModelFinder: fast model selection for accurate phylogenetic estimates. Nat Methods.

[28] Minh BQ, Nguyen MAT, von Haeseler A (2013). Ultrafast approximation for phylogenetic bootstrap. Mol Biol Evol.

[29] Kishino H, Hasegawa M (1989). Evaluation of the maximum likelihood estimate of the evolutionary tree topologies from DNA sequence data, and the branching order in hominoidea. J Mol Evol.

[30] Shimodaira H (2002). An approximately unbiased test of phylogenetic tree selection. Syst Biol.

[31] Martin DP (2005). A modified Bootscan algorithm for automated identification of recombinant sequences and recombination breakpoints. AIDS Res Hum Retrovir.

[32] Martin DP, Williamson C, Posada D (2005). RDP2: recombination detection and analysis from sequence alignments. Bioinformatics.

[33] Padidam M, Sawyer S, Fauquet CM (1999). Possible emergence of new Geminiviruses by frequent recombination. Virology.

[34] Smith JM (1992). Analyzing the mosaic structure of genes. J Mol Evol.

[35] Gibbs MJ, Armstrong JS, Gibbs AJ (2000). Sister-scanning: a Monte Carlo procedure for assessing signals in recombinant sequences. Bioinformatics.

[36] Drummond AJ (2012). Bayesian Phylogenetics with BEAUti and the BEAST 1.7. Mol Biol Evol.

[37] Rambaut A (2018). Posterior summarization in Bayesian Phylogenetics using tracer 1.7. Syst Biol.

[38] Kishino H, Miyata T, Hasegawa M (1990). Maximum likelihood inference of protein phylogeny and the origin of chloroplasts. J Mol Evol.

[39] Strimmer K, Rambaut A (2002). Inferring confidence sets of possibly misspecified gene trees. Proc Biol Sci.

[40] Andrews P (1992). Evolution and environment in the Hominoidea. Nature.

[41] Higham T (2014). The timing and spatiotemporal patterning of Neanderthal disappearance. Nature.

[42] Bravo IG, Alonso Á (2004). Mucosal human papillomaviruses encode four different E5 proteins whose chemistry and phylogeny correlate with malignant or benign growth. J Virol.

[43] Schiffman M (2005). The carcinogenicity of human papillomavirus types reflects viral evolution. Virology.

[44] Shah SD, Doorbar J, Goldstein RA (2010). Analysis of host–parasite incongruence in papillomavirus evolution using importance sampling. Mol Biol Evol.

[45] Longworth MS, Laimins LA (2004). Pathogenesis of human papillomaviruses in differentiating epithelia. Microbiol Mol Biol Rev.

[46] Bolatti EM (2016). Characterization of novel human papillomavirus types 157, 158 and 205 from healthy skin and recombination analysis in genus gamma-papillomavirus. Infect Genet Evol.

[47] Narechania A (2005). Phylogenetic incongruence among oncogenic genital alpha human papillomaviruses. J Virol.

[48] Rodríguez R (2012). M.d.R., et al., *Identificación de factores de riesgo para contraer virus del papiloma humano en sexoservidoras*. Rev Cuba Obstet Ginecol.

[49] Castoe TA (2009). Evidence for an ancient adaptive episode of convergent molecular evolution. Proc Natl Acad Sci U S A.

[50] Van Doorslaer K, McBride AA (2016). Molecular archeological evidence in support of the repeated loss of a papillomavirus gene. Sci Rep.

[51] Willemsen, A. and I.G. Bravo, Origin and evolution of papillomavirus (onco)genes and genomes. bioRxiv, 2018. 10.1098/rstb.2018.0303 PMC6501903 30955499

[52] Zanier K (2013). Structural basis for hijacking of cellular LxxLL motifs by papillomavirus E6 oncoproteins. Science.

[53] Suarez I, Trave G (2018). Structural insights in multifunctional papillomavirus Oncoproteins. Viruses.

[54] Puustusmaa M (2017). The enigmatic origin of papillomavirus protein domains. Viruses.

[55] Van Doorslaer K (2017). The papillomavirus episteme: a major update to the papillomavirus sequence database. Nucleic Acids Res.

